# The Gut Microbiota of Healthy Aged Chinese Is Similar to That of the Healthy Young

**DOI:** 10.1128/mSphere.00327-17

**Published:** 2017-09-27

**Authors:** Gaorui Bian, Gregory B. Gloor, Aihua Gong, Changsheng Jia, Wei Zhang, Jun Hu, Hong Zhang, Yumei Zhang, Zhenqing Zhou, Jiangao Zhang, Jeremy P. Burton, Gregor Reid, Yongliang Xiao, Qiang Zeng, Kaiping Yang, Jiangang Li

**Affiliations:** a Tianyi Health Sciences Institute (Zhenjiang) Co., Ltd., Zhenjiang, Jiangsu, China; b Departments of Biochemistry and of Applied Mathematics, Western University, London, Ontario, Canada; c Lawson Health Research Institute, London, Ontario, Canada; d Department of Gastroenterology, Affiliated Hospital of Jiangsu University, Zhenjiang, China; e Department of Orthopedics, Lanzhou Military General Hospital, Lanzhou, Gansu, China; f Gansu Provincial People’s Armed Police Corps Hospital, Lanzhou, Gansu, China; g School of Public Health, Peking University, Beijing, China; h Center for Disease Control and Prevention, Taicang, Jiangsu, China; i Wenci Hospital, Rugao, Jiangsu, China; j Department of Microbiology and Immunology, Western University, London, Ontario, Canada; k Health Management Institute, Chinese PLA General Hospital, Beijing, China; l Children’s Health Research Institute, London, Ontario, Canada; m Department of Obstetrics and Gynecology, Western University, London, Ontario, Canada; n Department of Physiology and Pharmacology, Western University, London, Ontario, Canada; Arizona State University

**Keywords:** 16S rRNA gene sequencing, DNA sequencing, compositional data, cross-sectional study, gut microbiota, healthy aging, microbiota

## Abstract

The microbiota of the aged is variously described as being more or less diverse than that of younger cohorts, but the comparison groups used and the definitions of the aged population differ between experiments. The differences are often described by null hypothesis statistical tests, which are notoriously irreproducible when dealing with large multivariate samples. We collected and examined the gut microbiota of a cross-sectional cohort of more than 1,000 very healthy Chinese individuals who spanned ages from 3 to over 100 years. The analysis of 16S rRNA gene sequencing results used a compositional data analysis paradigm coupled with measures of effect size, where ordination, differential abundance, and correlation can be explored and analyzed in a unified and reproducible framework. Our analysis showed several surprising results compared to other cohorts. First, the overall microbiota composition of the healthy aged group was similar to that of people decades younger. Second, the major differences between groups in the gut microbiota profiles were found before age 20. Third, the gut microbiota differed little between individuals from the ages of 30 to >100. Fourth, the gut microbiota of males appeared to be more variable than that of females. Taken together, the present findings suggest that the microbiota of the healthy aged in this cross-sectional study differ little from that of the healthy young in the same population, although the minor variations that do exist depend upon the comparison cohort.

**IMPORTANCE** We report the large-scale use of compositional data analysis to establish a baseline microbiota composition in an extremely healthy cohort of the Chinese population. This baseline will serve for comparison for future cohorts with chronic or acute disease. In addition to the expected difference in the microbiota of children and adults, we found that the microbiota of the elderly in this population was similar in almost all respects to that of healthy people in the same population who are scores of years younger. We speculate that this similarity is a consequence of an active healthy lifestyle and diet, although cause and effect cannot be ascribed in this (or any other) cross-sectional design. One surprising result was that the gut microbiota of persons in their 20s was distinct from those of other age cohorts, and this result was replicated, suggesting that it is a reproducible finding and distinct from those of other populations.

## Abstract (precis)

We report the large-scale use of compositional data analysis to establish a baseline microbiota composition in an extremely healthy cohort of the Chinese population. This baseline will serve for comparison for future cohorts with chronic or acute disease. In addition to the expected difference in the microbiota of children and adults, we found that the microbiota of the elderly in this population was similar in almost all respects to that of healthy people in the same population who are scores of years younger. We speculate that this similarity is a consequence of an active healthy lifestyle and diet, although cause and effect cannot be ascribed in this (or any other) cross-sectional design. One surprising result was that the gut microbiota of persons in their 20s was distinct from those of other age cohorts, and this result was replicated, suggesting that it is a reproducible finding and distinct from those of other populations.

## INTRODUCTION

Nobel Laureate Elie Metchnikoff is credited not only with providing insight into phagocytosis, but also with linking gut microbes and intake of fermented food to health and longevity (1). The search for healthy aging has been resurrected by the ability to identify a plethora of microbes at various body sites, particularly the gut, and to show that they influence and are influenced by health there and at distant sites (2, 3). This has led to investigations into the development of the gut microbiota throughout life, with some studies reporting a gradual change over time (4) based upon relatively small sample sizes (5–7). In a study of 728 female twins, 637 operational taxonomic units (OTUs) were associated with frailty, including *Eubacterium dolichum* and *Eggerthella lenta*, with *Faecalibacterium prausnitzii* less abundant (8). Since frailty is strongly associated with increasing risk of earlier mortality (9), it is important to characterize the gut microbiota along the continuum of age. The recent consensus is that, in aggregate, the diversity of the gut microbiota declines with age (10), although whether this is associated with healthy aging is controversial and the delineation of what is normal in different cohorts are still not clear. Thus, continued study of the gut microbiota in large and distinct cohorts is needed to identify and separate potential microbial biomarkers for age and frailty.

A study of 314 healthy Chinese subjects showed that ethnicity and lifestyle could be discriminated at the bacterial species level (11). The latter study led us to hypothesize that the gut microbiota is imprinted in early life and associated with longevity. We gained access to a large number of fecal samples from a variety of communities across the age continuum in China and used a compositionally coherent approach to examine the variance of the OTUs across all samples with age as a continuous variable for exploratory data analysis and compositional association and as a discrete variable for differential abundance analysis.

Our results show that the microbial composition of the healthy aged population is remarkably similar to that of younger adult cohorts and that the major differences between cohorts in microbial composition occur prior to age 30 years. While our cross-sectional cohort precludes the assignment of cause and effect, our results suggest that diet and lifestyle choices consistent with healthy aging even into the 10th decade of life include a healthy and diverse microbiota.

## RESULTS

The primary cohort of samples was collected from extremely healthy volunteers in three cities from Jiangsu Province in China. These cities, Zhenjiang, Suzhou, and Nantong, were located west of Shanghai on the Yangtze River. Table 1 contains the detailed geographic locations, age and sex distributions, and number of samples included in each group. Subjects under the age of 30 were included if their parents and grandparents lived to at least 80 years of age without major health problems that required surgery or long-term medication. Subjects aged 30 years old or older were included if the subject self-reported as being in extremely good health at the time of collection (see Materials and Methods). A secondary cohort was collected from young people undergoing military and police training in Lanzhou, a city in the north-central part of China. All metadata, tables of derived data, and code required to reconstitute all figures and analyses are publicly available at https://doi.org/10.6084/m9.figshare.4535660. The first analysis was performed on the data set with 883 samples collected from the general population. This data set included 577 operational taxonomic units (OTUs) that occurred at an abundance of greater than 0.1% in any sample and that occurred in at least 20% of the samples.

**TABLE 1:** Summary of sample groups and sites

Group	Age (yr)	No. female, male	Sampling location
Children			
Kindergarten	3–6	60, 43	Dagang Central Kindergarten, Zhenjiang City, Jiangsu Province; Taicang Weiyang Kindergarten, Suzhou City, Jiangsu Province; Taicang Huasheng Kindergarten, Suzhou City, Jiangsu Province
Primary school	8–12	90, 71	Dagang Central Primary School, Zhenjiang City, Jiangsu Province; Taicang Zhu Diwen Primary School, Suzhou City, Jiangsu Province
Middle school	13–14	65, 49	Taicang No. 2 Middle School, Suzhou City, Jiangsu Province
Adults			
Youth	19–24	55, 80	Taicang Chien-Shiung Institute of Technology, Suzhou City, Jiangsu Province
Middle age	30–50	66, 20	Taicang Xingda Can Co., Ltd., Suzhou City, Jiangsu Province
Elderly	60–79	43, 43	Taicang Xinhu Community, Suzhou City, Jiangsu Province
Centenarians	>94	142, 56	RuGao City, Nantong City, Jiangsu Province
Young soldiers	19–24	12, 200	Gansu police detachment training base and PLA training base in Lanzhou, Gansu Province

Initial exploration of the general population cohort by principal-component analysis (PCA) of centered log-transformed data (12) shows that the samples segregate with an observable but weak separation by age group (see “Samples” in Fig. S1 in the supplemental material).

10.1128/mSphere.00327-17.1 FIG S1  PCA plot of the initial data set. These two plots show the first two principle components of a singular value decomposition of the centered log ratio-transformed data for the data set comprising samples from people aged 3 to ≥100 years. The left panel shows a plot where each point is a sample, where the distance between points is proportional to the difference between samples. Samples are colored by their grouping, and the 75% data ellipses demonstrate that groups separate broadly by age cohort. The right panel shows the contribution of the OTUs to the separation of the samples. The distance and direction from the origin to the point representing an OTU are proportional to the standard deviation of the OTU in the data set. The distance between two OTUs is inversely proportional to their compositional association: points that are close together may have concordant abundances across all samples. The ability to directly interpret the plot is limited by the small proportion of variance explained. OTUs are colored gray if deemed uninteresting, red if they were part of a group where E(ρ) is >0.65, blue if the effect size was ≥1 for any pairwise comparison between groups, or magenta if both approaches deemed them to be of further interest. See the Materials and Methods section for the method used to calculate these values. Download FIG S1, TIF file, 1.7 MB. Copyright © 2017 Bian et al. 2017 Bian et al. This content is distributed under the terms of the Creative Commons Attribution 4.0 International license.

**FIG 1:**
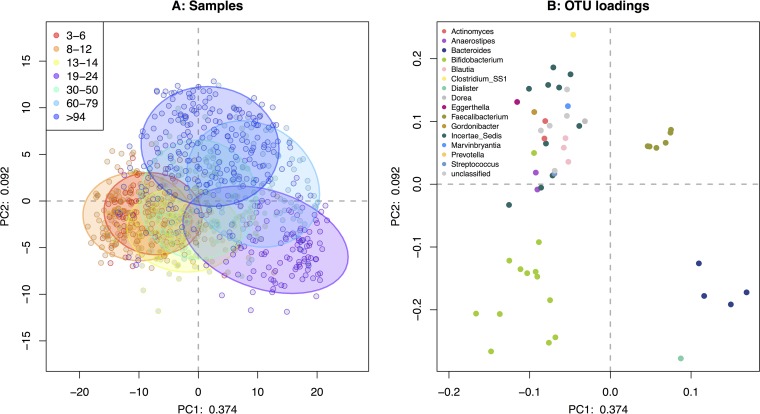
Compositional PCA plot of samples (A) and OTU loadings (B) for the initial data set. Only OTUs that had an absolute effect size difference between any two groups of ≥1 (24) or were compositionally associated with a ρ value of >0.65 (22) (Materials and Methods) are included in these plots. In panel A, each point is a sample and the distance between points is proportional to the multivariate difference between samples. Samples are colored by their age group membership, and the data ellipses encompass 75% of the points in a group (75% confidence interval [CI]). Panel B shows the loadings for panel A in the same coordinate space, which represent the contributions of the OTUs to the separation of the samples. In this plot, each point is an OTU (colored by its assigned taxonomic genus) and the distance and direction from the origin to the point representing an OTU is proportional to the standard deviation of that OTU in the data set. The distance between one OTU and another is inversely proportional to their compositional association: points that are close together may have concordant relative abundances across all samples. In comparing the two plots, we see, for example, that the 19- to 24-year-old age group (lower right quadrant in panel A) has a higher relative representation of *Bacteroides* (lower right quadrant in panel B). The ability to directly interpret the plot is limited by the proportion of variance explained (37.4% on the first component and 9.2% on the second component).

Figure 1 shows a compositional PCA plot of the samples and the associated loadings (13, 14) for the 62 OTUs that were judged to be the most explanatory of this data set, as outlined in the Materials and Methods; these OTUs are identified in the “OTUs” panel of Fig. S1. While there is significant overlap in the location of the samples from each group, the most extreme groups were the youngest (ages 3 to 6 and 8 to 12 years), the oldest (age >94 years), and, surprisingly, the 19- to 24-year-old group. The loading plot in Fig. 1B shows the OTUs responsible for the difference in location of the samples.

The finding that the 19- to 24-year-old group was divergent from the general population was reexamined by rerunning the analysis with the inclusion of a second cohort of 212 samples collected from military and police training groups (here the “young soldier” group): the entire data set in this case included 1,095 samples and 562 OTUs when filtered as for the general population cohort. The young soldier group included people who originated from various regions of China, were largely male, and again were between the ages of 19 to 24, inclusive. As shown in Fig. S2A in the supplemental material, the young soldier group clustered entirely and tightly within the extreme end of the 19- to 24-year-old group. An unsupervised clustering of the entire data set supported the general conclusion that the 19- to 24-year-old and young soldier groups and the >94-year-old and the 3- to 6-year-old groups were the most distinctive (Fig. S2B). The tight clustering of the young soldiers within the 19- to 24-year-old group was likely because the young solider group members were selected to be very healthy and very active, and all were housed together in two distinct common environments.

10.1128/mSphere.00327-17.2 FIG S2  Multivariate exploration of the data set. Panel A shows that samples from individuals that are near 20 years old separate from all other samples even when the young soldier samples are included in this data set; these are the 19- to 24S samples in the legend. As in Fig. 1, samples are filtered to include all taxa that occur with a minimum frequency in any sample of 0.001 and that occur in at least 20% of samples. Panel B shows an unsupervised clustering approach using the Aitchison distance (Euclidian distance of the clr values) and Ward.D2 clustering, including all OTUs. Samples are colored by group. Essentially, there are three large partitions. One was composed of the majority of members of the 3-to 6-year-old and 8-to-12-year-old groups, one composed of both 19- to 24-year-old groups, and one composed of the remainder. This last group is heterogeneous and likely results because the 13- to 14-year-old and 30- to 50-year-old groups are closest to the center of the data set. Panel C is a PCA plot of the entire data set, including only the OTUs that are different or compositionally associated. Samples from individuals that are in the 19- to 24-year-old groups are separate from all other samples. Panel D shows the loading plot for PCA in panel C and includes only the subset of OTUs that are different between groups with an effect size of ≥1 or that are compositionally associated. Download FIG S2, PDF file, 0.2 MB. Copyright © 2017 Bian et al. 2017 Bian et al. This content is distributed under the terms of the Creative Commons Attribution 4.0 International license.

The young soldier group comprises people from a military training center in Lanzhou and from a separate police training facility in Lanzhou. The different groups were enrolled in distinct training regimens and did not mess together. Inclusion of only the 108 OTUs with the greatest effect or that were compositionally associated in this entire data set provided a similar ordination (Fig. S2C and D) to that seen when the young soldier group was excluded (Fig. 1A).

All pairwise group comparisons were distinct when examined by permutational multivariate analysis of variance (PERMANOVA [*P* < 0.001]) using the Aitchison distance (15). Examination of the PERMANOVA results showed that the 19- to 24-year-old and young soldier groups were more similar to each other than to any other group and that the group differences were otherwise small. Thus, the low *P* values likely reflect the large number of samples and not necessarily a large difference between all groups (see Table S1 in the supplemental material). Furthermore, we observed that the young soldier group had the smallest within-group dispersion and the >94-year-old group the greatest (Table S1).

10.1128/mSphere.00327-17.8 TABLE S1  Table of PERMANOVA test of location values calculated using the Vegan Adonis function, using the Aitchison distance (method=euclidian) using clr-transformed values. The “R2” value is the proportion of the variance that is estimated to be caused by a location (difference in mean value) difference. “F.Model” represents the *F* statistic values, and “P” is the estimated PERMANOVA *P* value. Only the youth (“you”) and young soldier (“ys”) groups are strongly different from their bracketing cohorts. “MCD” is the median Aitchison distance of each sample to the group A centroid, and “IQR” is the interquartile range of those distances. The final row is a placeholder to show the MCD and IQR for the >94-year-old age group (cent). Values were calculated using the betadisper function in the vegan R package (40). The young soldier group is less disperse than the others, and there is a tendency for the two oldest groups, aged 60 to 79 (eld) and >94 (cent), to have greater dispersion. Download TABLE S1, DOCX file, 0.01 MB. Copyright © 2017 Bian et al. 2017 Bian et al. This content is distributed under the terms of the Creative Commons Attribution 4.0 International license.

The exploratory data analysis shown in Fig. 1 and Fig. S2 revealed several interesting patterns in the microbiota composition for this large age-segregated data set. First, OTUs assigned to the *Bifidobacterium* genus appeared to be relatively enriched in the youngest groups and relatively depleted in the oldest groups. Conversely, OTUs assigned to the genera *Dorea*, *Clostridium insertae sedis* (IS) and *sensu strictu* 1 (SS1), *Marvinbryantia*, and, to a lesser extent, members of the *Prevotella* genus appeared to be relatively enriched in the samples collected from older subjects.

The age gradients and segregation of the 19- to 24-year-old and young soldier groups were very robust to data manipulations (see Fig. S3 in the supplemental material). In particular, the same separation between groups was observed when rare OTUs were excluded (minimum relative abundance in any sample of 1%) or common OTUs were excluded (maximum relative abundance in any sample of 2%). We thus concluded that the structure of the data was a function of the entire microbial ecosystem and was not driven by either rare or abundant taxa. This is in contrast to other studies where the separation of particular groups was driven by only a few relatively abundant taxa (16, 17).

10.1128/mSphere.00327-17.3 FIG S3  Filtering by relative abundance does not change the conclusions. The left plot shows the data filtered to a minimum abundance in any sample of 1%, and the right plot shows the data filtered to a maximum abundance in any sample of 2%. This demonstrates that the split between groups is intrinsic to the composition of the microbiota as a whole and is not driven by a preponderance of rare or abundant OTUs in the groups. Download FIG S3, TIF file, 1.4 MB. Copyright © 2017 Bian et al. 2017 Bian et al. This content is distributed under the terms of the Creative Commons Attribution 4.0 International license.

Examination of confounding factors suggested that the age groups were distinct from each other and that subsetting the age groups by metadata failed to reveal any substantial variance within groups that could be attributed to the metadata (see Fig. S4 in the supplemental material). We conclude that no other metadata better explained the data.

10.1128/mSphere.00327-17.4 FIG S4  Exploration of the data set suggests that sample group is likely the major explanatory factor. Download FIG S4, TIF file, 1.8 MB. Copyright © 2017 Bian et al. 2017 Bian et al. This content is distributed under the terms of the Creative Commons Attribution 4.0 International license.

However, while the overall pattern of group separation was similar, the percentage of variance explained was different for the ordination when only the 562 male-derived samples or the 533 female-derived samples were examined. Including samples from females only, principal component 1 (PC1) explained 11.3% of the variance, while PC1 explained 19.4% in the male-only samples. The percentages of variance explained on the second PCA axis were essentially the same at 7.4% and 6.4%.

**FIG 2:**
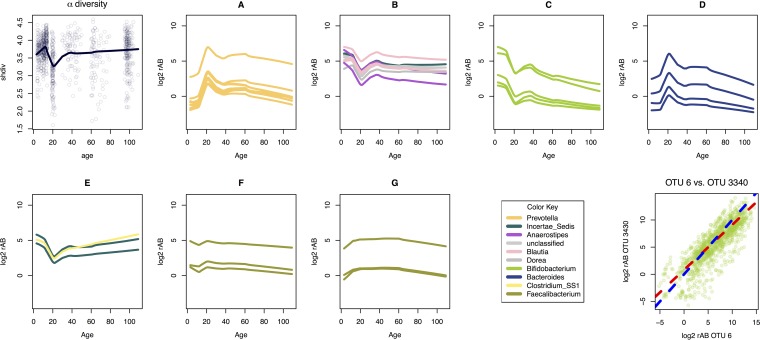
Exploration of the data set with age as a continuous variable. The α diversity panel plots Shannon’s diversity (shdiv) across the age range (*x* axis). Each point is an individual sample, and the black line is the Loess line of best fit. Panels A through G represent clusters of concordant OTUs with an expected ρ value cutoff of >0.65; this metric provides a measure of the constancy of the ratio between OTUs and is a replacement for correlation (18, 22, 23). Each line in a cluster plot is the Loess line of best fit for the clr relative abundance (rAB on the y axis) of an individual OTU across age (*x* axis). A 0 value indicates that the relative abundance of an OTU is equal to the mean log_2_ relative abundance of all OTUs, while a positive or negative value indicates relative abundances greater or less than the mean log_2_ relative abundance, respectively. OTU lines of best fit are colored according to the genus that the OTU is classified into according to the key. Note that most of the clusters contain OTUs related by the same genus (A, C, D, F, and G). The lines of best fit suggest approximately equal ratios between the cluster members across the age range; however, this must be investigated further (22, 23), as shown in the last panel. For demonstration, the relative abundances between one pair of concordant OTUs from cluster C is plotted in the bottom right panel (OTU 6 versus OTU 3340). These two OTUs are the two relatively most abundant OTUs in cluster C (top two lines), and they have an expected ρ value of 0.8. The slope of association shown in the red line is 0.82. The blue line shows the ideal slope of 1 (22, 23). The Pearson correlation coefficient is 0.83. Table S2 contains the slope and correlation information for all pairwise correlated OTUs.

The data were next examined by plotting age as a continuous variable versus Shannon’s diversity, and the results are shown in the α diversity panel in Fig. 2. The range of values is rather narrow but exhibited a modest peak diversity near age 10 and a slight minimum near age 20. The pattern of Shannon’s diversity is not an artifact of read depth since examination of samples from all ages with a narrow read depth band exhibited the same pattern, and α diversity and read depth were not found to be correlated (see Fig. S5 in the supplemental material).

10.1128/mSphere.00327-17.5 FIG S5  Exploration of α diversity in the groups shows that diversity is correlated with the age gradient and that the read count (RC) is not associated with Shannon’s diversity (ShDiv). Download FIG S5, TIF file, 2.2 MB. Copyright © 2017 Bian et al. 2017 Bian et al. This content is distributed under the terms of the Creative Commons Attribution 4.0 International license.

10.1128/mSphere.00327-17.9 TABLE S2  Summary of E(ρ) data. Each OTU pair has an expected value of ρ of >0.65; however, this measure is a single value that encapsulates both the slope and Pearson’s correlation of an association (18, 22). The “slope” and “corr” columns show the slope of the association and Pearson’s correlation coefficient. Slopes near 1 and correlations near 1 indicate a better fit to the model of compositional association. Download TABLE S2, DOCX file, 0.02 MB. Copyright © 2017 Bian et al. 2017 Bian et al. This content is distributed under the terms of the Creative Commons Attribution 4.0 International license.

Identification of correlated features in compositional data is notoriously difficult (18–22), and in particular Pearson’s or Spearman’s correlations give many false-positive associations (18). Thus, we used an expected value of ρ metric (22) to identify clusters of coassociated OTUs in the data set. This strength of association approach identifies OTUs where both the direction and magnitude of variance are similar in the multidimensional data set. In a multivariate compositional sense, the metric is measuring both the slope of the correlation of the centered log ratio (clr)-transformed values and the correlation itself. A slope of 1 and a correlation of 1 are preferred (18, 22, 23). The ρ metric coupled with a Bayesian estimation of OTU relative abundance (22, 24) has the advantage of being agnostic to the level of sparsity in the data, unlike other recent approaches that depend on this sparsity (19, 20).

Panels A to G in Fig. 2 show the relative abundance of OTUs assigned to one of 9 genera (plus unknown genera) that had an expected ρ value of >0.65, and where the cluster size was larger than 2, plotted against age as a continuous variable. For reference, the last panel in Fig. 2 shows the correlation and slope of one pair of OTUs. The correlation and slope for all pairs of OTUs identified are given in Table S2 in the supplemental material.

This approach identified seven distinct clusters, and most clusters contained OTUs classified into the same genus. It is likely that the most strongly associated groups include predominantly members of the same genus because different members of the same genus have similar growth requirements, limitations, and interactions.

Clusters F and G were relatively constant across all samples and were composed of OTUs identified as being members of the *Faecalibacterium* genus, suggesting that they are members of the core microbiota in this population. This result contrasts with the findings of Biagi et al. (25), who observed that the OTUs assigned to the genus *Faecalibacterium* as a whole were relatively less abundant in centenarians than in younger persons.

Clusters A to E showed a large change in relative abundance in the 19- to 24-year-old group compared to all other age groups. In particular the relative abundance of OTUs assigned to the genera *Prevotella* and *Bacteroides* (clusters A and D) were greatest in the 19- to 24-year-old group, and OTUs classified in both these genera were relatively rarer in people younger than 20. Members of the *Bacteroides* and of the *Bifidobacterium* genera were, in general, relatively least abundant in the samples from the oldest subjects. The *Prevotella* spp. in cluster A tended to remain relatively constant after age 30, but decreased somewhat in the oldest subjects.

Clusters B, C, and E showed a local minimum in relative abundance near age 20. Clusters B and E exhibited an otherwise somewhat constant relative abundance, likely reflecting a set of taxa that are part of the core microbiota displaced by the OTUs assigned to the *Prevotella* and *Bacteroides* genera in the 19- to 24-year-old and young soldier groups. In contrast, the members of cluster C, composed entirely of OTUs assigned to the *Bifidobacterium* genus, were relatively less abundant in the 19- to 24-year-old groups than in the age groups that immediately surround the 19- to 24-year-old and young soldier groups. Members of cluster C exhibited a continuous reduction in relative abundance from age 30 onwards compared to the relative abundance in the younger subjects.

Examining the Shannon’s diversity plot, the local minimum in α diversity near age 20 may be caused by members of the *Prevotella* and *Bacteroides* genera achieving relative dominance in this age group, thus displacing (or appearing to displace) the remainder of the members of the ecosystem and reducing the overall diversity of the system.

**FIG 3:**
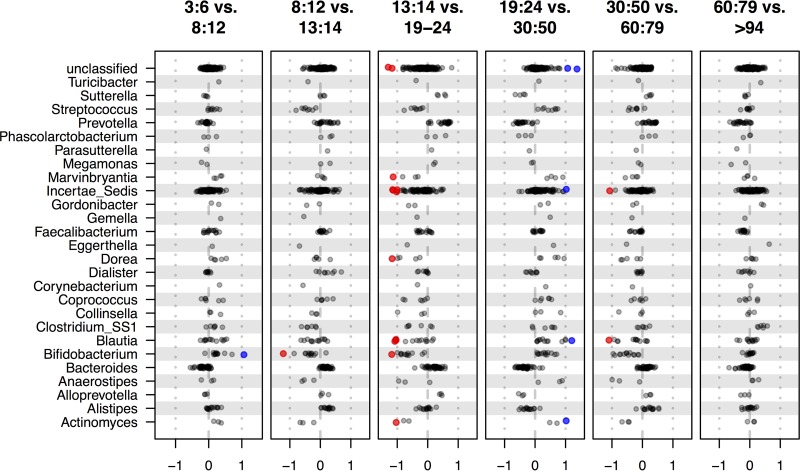
Differential relative abundance of major taxa for successive pairwise comparisons. All possible comparisons of cohorts revealed 102 OTUs that were reproducibly different between at least one pair of cohorts. These were grouped into 26 classified genera and one set containing all unclassified OTUs. The plots show all OTUs in these 26 genera for pairwise comparisons between each successive age group pair: 3 to 6 versus 8 to 12, 8 to 12 versus 13 to 14, 13 to 14 versus 19 to 24, 19 to 24 versus 30 to 50, 30 to 50 versus 60 to 79, and 60 to 79 versus >94. Each comparison plot shows a point for each OTU binned by genus with the log_2_ standardized difference, the “effect” measure determined by ALDEx2 (24, 39), between the two groups on the *x* axis. Points are colored as red or blue if they have an effect size of ≥1 for the comparison. An effect size greater than 1 indicates that the OTU will be reliably found to have a greater difference between groups than dispersion within either group (24). Equivalent plots for comparisons at different taxonomic levels are shown in Fig. S6.

Figure 3 shows the standardized effect size differences calculated by the ALDEx2 R package (see Materials and Methods) between each successive group with the OTUs binned by genus. It is striking that the majority of the OTUs in the majority of genera exhibit strikingly concordant differences in relative abundance, even if those differences do not reach an effect size greater than 1. This amplifies the results shown in Fig. 1 and 2, where only a subset of OTUs were displayed. A multitude of small genus-wide differences in relative abundance will have cumulatively large effects on the composition of the microbiota. To our knowledge, no other study has examined the association between OTU-level changes and genus- or other taxonomic-level changes in such a granular manner.

10.1128/mSphere.00327-17.6 FIG S6  Differential relative abundance of major taxa for successive pairwise comparisons grouped by family (top), class (middle), and phylum (bottom). All possible comparisons of group revealed many OTUs that were reproducibly different between at least one pair of cohorts. These were grouped into 26 genera and one set of unknown genera. Points are colored as red or blue if they have an effect size of ≥1 as determined by ALDEx2 for the comparison. Download FIG S6, TIF file, 2.8 MB. Copyright © 2017 Bian et al. 2017 Bian et al. This content is distributed under the terms of the Creative Commons Attribution 4.0 International license.

Not surprisingly, the association between OTU-level and taxonomic rank-level changes becomes less obvious with higher taxonomic rank, although large-scale trends can still be observed (see Fig. S6 in the supplemental material). At the phylum level, OTUs classified in the *Tenericutes* and *Proteobacteria* were relatively similar across all age groups. In contrast, the bulk of the *Firmicutes* exhibited large-scale difference, being relatively less abundant in the 19- to 24-year-old and 60- to 79-year-old groups, although there were many OTUs that exhibited trends away from the majority. More clearly, individual OTUs in the *Actinobacteria* and the *Bacteriodetes* phyla tended to have altered relative abundances that were more closely allied with the difference observed for all the OTUs in the phylum. Thus, alterations in the relative abundance of particular members of the *Actinobacteria* or *Firmicutes* phylum appeared to be compensated for by differences in relative abundances of OTUs in the *Bacteriodetes* or *Firmicutes* phylum or both phyla.

Second, we found that entire genera, and other taxonomic levels, are relatively increased or decreased in different age groups, indicating that the behavior observed for a small number of OTUs in Fig. 2, is often genus-wide. For example, OTUs in the genus *Blautia* displayed little difference in the 3- to 14-year-old age range, but are relatively depleted in the 19- to 24-year-old group, become relatively more abundant in subjects older than 30, and then become relatively depleted again in subjects older than age 60. A similar pattern is observed for *Bifidobacterium*, except that the earliest evidence of relative depletion occurs in the transition from primary to middle school (8- to 12-year-olds versus 13- to 14-year-olds). In contrast, the bulk of the OTUs in the genera *Prevotella* and *Bacteroides* become relatively more abundant in all successive age groups until age 20, then relatively rarer in the 30- to 50-year-old group, relatively more abundant in the 60- to 79-year-old group, and relatively rarer again in those >94 years old.

## DISCUSSION

The present study identified a distinct gut microbiota in a 19- to 24-year-old cohort from the general population that has not been observed in large-scale analyses of other populations (25–27) and may be unique to this healthy cohort in China. This observation may result from an altered diet, altered energy requirements, or an unknown cohort effect, although if the latter, it must have occurred countrywide as the same effect was observed in a population of university age students from Jiangsu Province and from police and military recruits originating from all provinces in China.

There are three possibilities that can explain the data in this cross-sectional study. First, it is tempting to speculate that the patterns of occurrence can be interpreted as a trajectory that is established early on and reversed or disrupted in the 19- to 24-year-old cohort. Second, it is possible that the patterns observed represent a cohort effect, whereby each age group exhibits a pattern set up from a shared diet or environment. Third, it is possible that the differences between age groups can be accounted for by a survivor bias. This final explanation may be particularly important in the oldest age groups, which differed little from younger subjects. A lifelong longitudinal study would be required to distinguish between these three conjectures.

While the vast majority of samples were collected in Jiangsu Province, we believe that many of the observations will translate to other parts of China, in particular those regions with similar demographics, history, and cuisine. This is illustrated by the striking similarity between the 19- to 24-year-old group collected largely from Jiangsu Province and the young soldier cohort that was collected from two distinct groups of subjects of diverse origins in China. Importantly, the samples from the young soldier cohort were collected in a different way than for the remainder of the samples, yet the 19- to 24-year-old college students and young soldier samples cluster together on PCA plots, by unsupervised clustering, and had a small variance in the PERMANOVA analysis. Thus, given that the data from the 19- to 24-year-old group from Jiangsu Province are potentially generalizable to other people of similar ages from across China, it is possible that the data for other age groups may be similarly generalizable, at least in broad outline.

These data were analyzed using a compositionally coherent approach which examined the variance of the OTUs across all samples with age as a continuous variable for exploratory data analysis and for compositional association and as a discrete variable for differential abundance analysis. This unified approach gave consistent results across all three methods, which is in contrast to more standard approaches such as weighted Unifrac or Bray-Curtis dissimilarity, where the ordination or clustering is driven by the most abundant taxa, but the most differentially abundant taxa are often those that are rarest in the data set (17). We noted that an exploration of the data by nonmetric multidimensional scaling ordination identified the component 1 but not the component 2 separation, suggesting that the separation of the 19- to 24-year-old subjects was the most robust signal and that the compositional approach is more sensitive than a popular nonparametric ordination approach (see Fig. S7 in the supplemental material).

10.1128/mSphere.00327-17.7 FIG S7  Examination of the entire data set using nonmetric multidimensional scaling (NMDS) shows that the ordinations are broadly similar in that the two most extreme groups are the samples from the youngest group and the 19- to 24-year-old group. The separation between the >94-year-old group and the others is less pronounced based on this method, which uses only rank and not relative abundance information. The relationship is not linear at low levels of dissimilarity. Download FIG S7, TIF file, 2.2 MB. Copyright © 2017 Bian et al. 2017 Bian et al. This content is distributed under the terms of the Creative Commons Attribution 4.0 International license.

The diversity of gut microbial ecosystems across the healthy life span is somewhat controversial, with some reporting a decline in diversity with age in the elderly, especially the frail elderly (8, 10, 27), and others reporting that diversity either does not change or increases in the healthy elderly (8, 41). Our analysis, with a very large cohort containing all age groups from 3 to >100, where the participants are all either very healthy or from a family with a very healthy family history, suggests that the answer depends on which group with which the healthy elderly are compared: diversity increases dramatically relative to healthy 20-year-olds, but declines relative to those in aged 13 to 14, and appears to increase slightly compared to those between 30 and 79 years old. In addition, the >94-year-old group had a larger β diversity than did younger groups; thus, the small sample size of healthy aged people could result in spurious observations. Nevertheless, despite the relatively constant microbial composition in all age groups, there are reproducible differences in the microbiota composition between the age groups.

The largest differences in OTU abundance were found between groups early in life and around age 20, as well as in the extremely healthy elderly. There are large differences in relative abundance of the OTUs of many genera between subjects aged 19 to 24 and younger subjects. If we take the view that this is a cohort effect, we could conclude that members of multiple genera form a minimum or maximum relative abundance near age 20 (Fig. 3). This suggests that a change in lifestyle (e.g., leaving home for university or jobs) or physiology (e.g., levels of sex steroid hormones) in the postteen years is an important determinant of the observed gut microbiota. Indeed, the observed difference in microbiota in the 19- to 24-year-old groups does seem contemporaneous with the rapid rise in plasma levels of sex steroid hormones in males and females experienced at this stage in life, with testosterone in males and estradiol in females peaking around this age (28). These were not investigated in this cohort, but in animal models, the ability of microbes to regulate hormones and for them to change microbial diversity has been demonstrated (29). These differences were found to be more profound in extremely healthy and vigorously active young soldier cohort. Thus, we cannot rule out that either a difference in caloric intake or some other environmental or countrywide historical factor is the cause of the difference between the 19- to 24-year-old subjects and the others.

Enterotypes proposed by Arumugam (16) were not identified in this data set: in fact, the relative abundances of members of the *Prevotella* and *Bacteroides* genera are largely concordant across the age range. This is not surprising given that the occurrence of enterotypes relies on the dominance of one or more taxa in the data set (17). The compositional analysis that does not rely on abundance coupled with the relatively large number of samples and of taxa and the richness of the Chinese diet may account for the lack of enterotypes.

The OTUs in several genera were relatively constant across all age ranges, with members of the *Faecalibacterium* genus being most constant and some members of the *Blautia*, *Clostridium* I, *Anaerostipes*, *Dorea*, and *Turcibacter* genera being more variable (Fig. 2). These may form a core microbiota for this cohort and perhaps for residents of China in general, as future studies will determine.

The microbiota composition of the centenarian cohort was remarkably similar to those of all members of the cohort over the age of 30, bar a few small changes, in the 30- to 50-year-old cohort that were carried through to elder years. This may reflect a selection bias in our sample cohort for the very healthy elderly. Nevertheless, it is interesting that the very healthy >94-year-old cohort has been able to maintain a microbiota similar to that of healthy younger people—perhaps by staying in one place and consuming the same type of food. However, other host and environmental factors were not investigated.

Several methodological limitations of the study must be acknowledged. First, only limited participant metadata were collected. Second, we did not conduct negative-control DNA extractions or amplifications, although it is unusual for fecal samples to be of low biomass. Third, block randomization of samples was not conducted: although samples from every age group were in each processed batch, they were not randomized or blocked. Finally, the set of participants was self-selected and all personal information was self-reported.

### Conclusions.

The results suggest that if you live to be 100 and in perfect health in China, your microbiota will likely appear to be relatively similar to that from a person in their mid-30s. Whether this is cause or effect is unknown, but it suggests that resetting an elderly microbiota to that of a 30-year-old might help promote health, if the microbiota is outside the norm. This study showed the practicality and power of a compositional data analysis paradigm, where ordination, differential abundance, and correlation can be analyzed in a unified and robust framework.

## MATERIALS AND METHODS

### Enrollment and exclusion criteria for the study.

Volunteers were asked to fill out a self-reporting health information questionnaire that included information on the inclusion and exclusion criteria described below. The age and detailed geographic locations of all persons who contributed samples are included in Table 1. These were divided into eight groups by age and are referred to by their age range in years (Table 1): kindergarten students, 3 to 6; primary school students, 8 to 12; middle school students, 13 to 14; college students, 19 to 24; soldiers and police recruits, 19 to 24; middle-aged, 30 to 50; elderly, 60 to 79; and centenarians, at least 94.

Only subjects who self-reported as having a personal and family history of extreme health (based on the self-reported questionnaires) were included in this study. Inclusion criteria were nonsmoker, teetotaler, mood was stable (self-assessed/reported), absence of any diseases, no prescription medication and antibiotics for the past 3 months (including birth control pills), no personal and family disease history (such as cardiovascular, gastrointestinal, metabolic, neurological/mental and respiratory diseases, as well as cancers), and parents and grandparents are all alive or passed away after 80 years of age. This last criterion was not applied to subjects recruited older than 31 years of age. These stringent criteria excluded between 97% and 99% of potential volunteers depending on age. The volunteers in the young soldier category were chosen with all the criteria listed above and the following two additions: first, they had passed the standard military entrance medical examination, and second, their grandparents lived to be at least 85 years.

### Description of informed consent and ability to use data for publication.

Before sampling, all volunteers were informed about the purpose of this study and signed an informed consent form, which included provision of data acquired by examination of the samples they provided. The study was approved by the University of Jiangsu Affiliated Hospital Ethics Committee for Biomedical Research (Zhenjiang City, Jiangsu Province, China).

### Collection methods for each cohort.

Whole fecal samples were collected without preservatives following a standard operating procedure (SOP) described below. A sampling package was sent to subjects who met the inclusion criteria, and a collecting site was established nearby, with an icebox that was checked hourly. All samples were transferred to Tianyi Health Sciences Institute (Zhenjiang) Co., Ltd., in the icebox within <3 h of collection, mixed, aliquoted, and stored at −80°C. We used the same SOP with minor variations for the young soldier and >94-year-old groups. For the young soldiers’ samples, each subject collected a fecal sample following the instructions provided and immediately placed the sample outdoors where the temperature was lower than 0°C. Samples were aliquoted and frozen at 8 a.m. the morning after collection. For the subjects in the >94-year-old group, the sampling package was taken to their home and retrieved within 2 h of collection, and the samples were then aliquoted at WenCi Hospital. Samples were frozen at −80°C after aliquoting.

### DNA isolation methods.

DNA was extracted from all samples using the PowerSoil DNA Isolation kit (Mo Bio Laboratories, Inc.) following the manufacturer’s protocol, with modifications as outlined in the Earth Microbiome Project (version 4_13). DNA was quantified using a PicoGreen double-stranded DNA (dsDNA) reagent kit (Invitrogen; Paisley, United Kingdom) with a Molecular Devices SpectraMax microplate reader (Molecular Devices, Sunnyvale, CA). DNA samples were stored at −20°C until further processing.

The V4 region of the bacterial 16S rRNA gene was amplified by PCR using in-line barcodes coupled with the Earth Microbiome V4 primer sequences 515f and 806r (30), with a 4-nucleotide (nt) random sequence pad between the Illumina adapter and barcode sequence. The inline-used barcodes were CCAAGGTT, AAGGTTCC, GGTTCCAA, TTCCAAGG, CCTTGGAA, TTGGAACC, GGAACCTT, AACCTTGG, CTACTACC, GATGATGG, TCGTCGTT, AGCAGCAA, CTACCCTA, GATGGGAT, CGTTTCGT, GCAAAGCA, TTAACCGG, and AACCGGTT. This strategy permits combinatorial barcodes to be used to uniquely identify each sample in each run (30). A standard cycling procedure was used (2 min at 95°C, followed by 25 cycles of 1 min at 95°C, 1 min at 55°C, and 1 min 72°C, with a final 5 min at 72°C and hold at 4°C) using primers 515F (5-barcode-GTGCCAGCMGCCGCGGTAA-3′) and 806R (5′-barcode-GGACTACHVGGGTWTCTAAT-3′).

Amplicons were purified using the Qiagen QIAquick PCR purification kit (Qiagen; Düsseldorf, Germany) according to the manufacturer’s instructions and quantified using PicoGreen dsDNA reagent kit (Invitrogen; Paisley, United Kingdom). Purified amplicons were pooled in equimolar amounts, and the amplicon size was determined by an Agilent 2200 bioanalyzer.

### DNA sequencing methods.

The pooled product was paired-end sequenced with a 600 cycle kit on the Illumina MiSeq platform (Illumina, Inc., San Diego, CA) according to standard protocols.

### Postsequencing processing.

A brief description of the pipeline has been published previously (31), with the SOP and all software required available at http://github.com/ggloor/miseq_bin. Sequences were processed using a standardized pipeline (see Text S1 in the supplemental material). Reads were overlapped using PandaSeq v2.5 (32), and any reads that contained ambiguous positions (an N in either strand) were removed. Reads in each run were demultiplexed and assigned a name that uniquely reflected the sample identifier and barcode. Demultiplexed reads from all samples were pooled into one file and collapsed into individual sequence units (ISUs). Note that the ISU clustering step is extremely memory intensive and was conducted on a server with 256 Gb RAM. ISUs were ordered by abundance and were used for open reference OTU picking by USEARCH with a *de novo* chimera filtering step (33, 34). The OTUs occurring in each sample were tabulated, with singleton OTUs and those rarer than 0.1% in any sample excluded, resulting in an initial data set containing 1,514 OTUs apportioned across 1,095 samples. This table is contained on the FigShare site as tab-separated plain text in SupplementaryCountTable.txt and forms the basis of the analyses described below.

10.1128/mSphere.00327-17.10 TEXT S1  The exact workflow to generate the table used for 16S rRNA gene sequence analysis. Download TEXT S1, DOCX file, 0.02 MB. Copyright © 2017 Bian et al. 2017 Bian et al. This content is distributed under the terms of the Creative Commons Attribution 4.0 International license.

### Methods of analysis.

The data from high-throughput sequencing are relative abundance data and thus contain only information regarding the relationships or ratios between taxa (13–15, 18). Thus, we adopted a compositional data (CoDa) analysis approach (35) that examines the ratios between OTUs. The full workflow is contained on the FigShare site, but in brief, we did the following. For exploratory analysis, zero count OTUs were replaced by an imputed value using the count zero multiplicative method from the zCompositions R package (36). The centered log ratio (clr) transform was applied to the zero replaced data set, and the data were subsequently used as input for a singular value decomposition (SVD). This approach returns data where the samples are separated by the variance in the OTUs rather than by differences in abundant OTUs (14, 15). Initial exploration of the data was conducted by using PCA plots to explore the SVD output (12).

When conducting quantitative analyses, we used the clr-transformed posterior distribution of the data generated by 128 Monte Carlo replicates drawn from a Dirichlet distribution (13, 24, 37). All analyses report the expected value of the test statistic (22). Differential abundance tests were conducted with the ALDEx2 v1.6.0 Bioconductor package (24, 37), and we report those taxa that have an expected effect size difference of ≥1, since effect size measures are more reproducible than are *P* values (38). Correlation analyses were done using a symmetric modification of the ρ metric (22), which measures the variance in the ratios between OTUs. OTUs with a low ratio variance are said to be compositionally associated. The ρ metric (23) was calculated as an expected value across clr-transformed Monte Carlo Dirichlet replicates (22, 24) generated by the aldex.clr function, and values of >0.65 were taken as indicating association between pairs of OTUs.

The data were further subsetted to include only OTUs that had an expected effect size of greater than 1 in any pairwise age group comparison or had an expected compositional association value of E(ρ) > 0.65.

### Ethics approval and consent to participate.

This study was approved by the Affiliated Hospital of Jiangsu University Biomedical Research Ethics Committee (Zhenjiang, Jiangsu, China).

### Consent for publication.

Sample identities are anonymous, and no identifying information is available for the participants.

### Accession number(s).

Demultiplexed raw reads are being made available through the SRA database under accession no. SRP107602.

### Availability of data.

In the interests of reproducibility, all metadata R scripts and tables of derived data are publicly available at https://doi.org/10.6084/m9.figshare.4535660, as is the code required to reconstitute all figures.
