# Supplementary figures and images for: The Gut Microbiota of Healthy Aged Chinese Is Similar to That of the Healthy Young
Source: mSphere. 2017 Sep 27;2(5):e00327-17. doi: 10.1128/mSphere.00327-17 (PMC5615133; doi:10.1128/mSphere.00327-17)

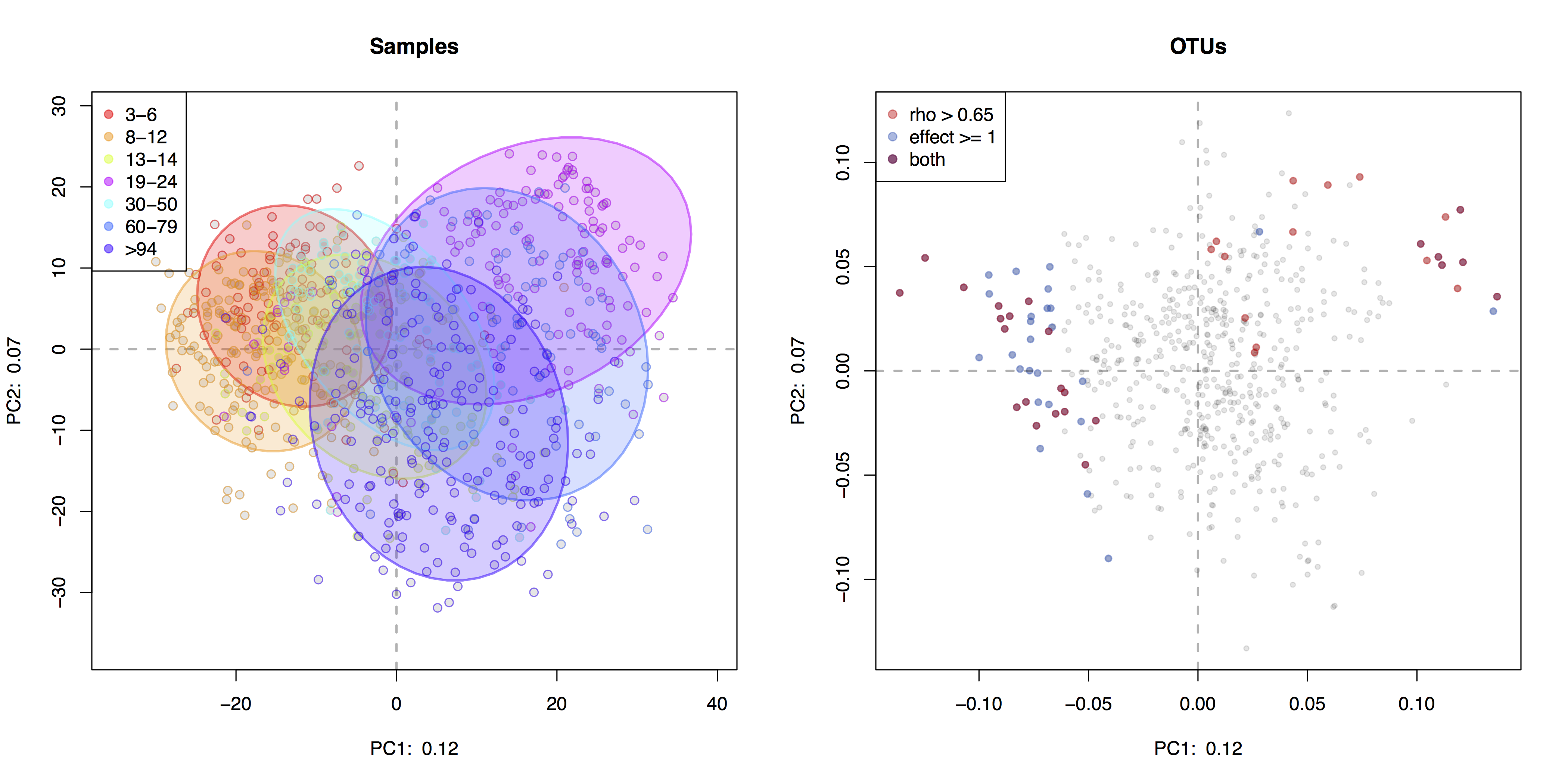

Supplement: FIG S1 [file sph005172374sf1.tif]

**A: All sample PCA with all OTUs**

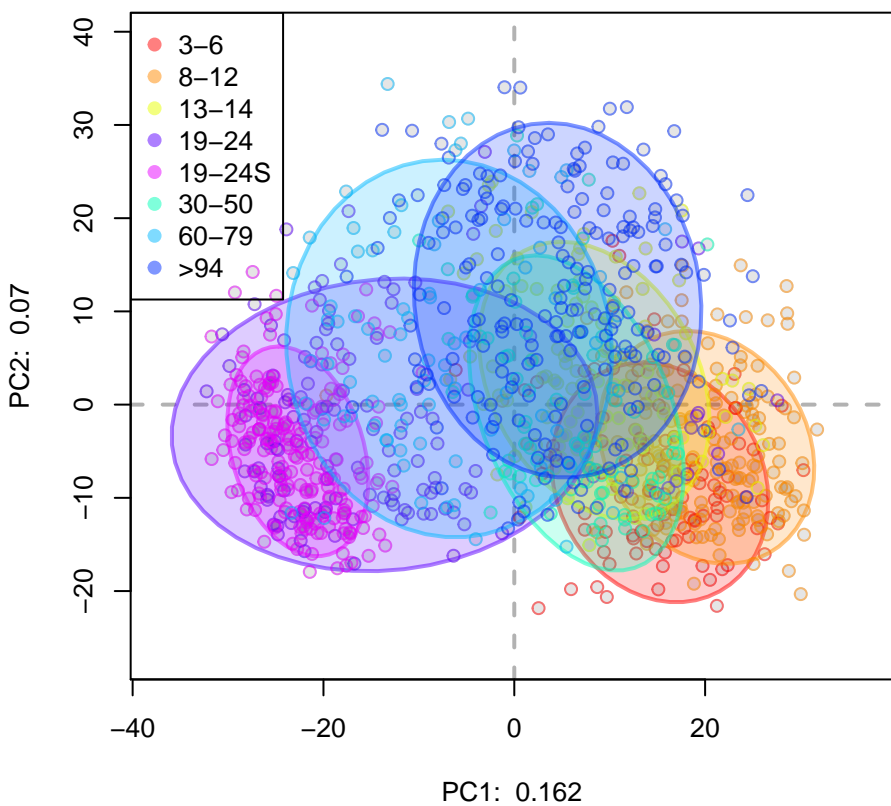

**B: dendrogram**

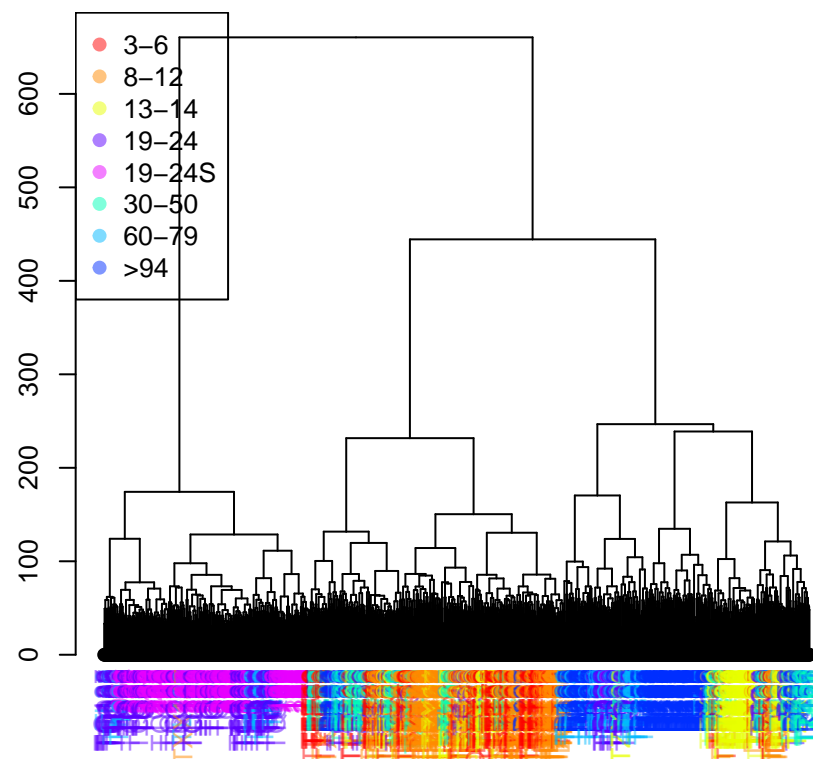

**C: Samples**

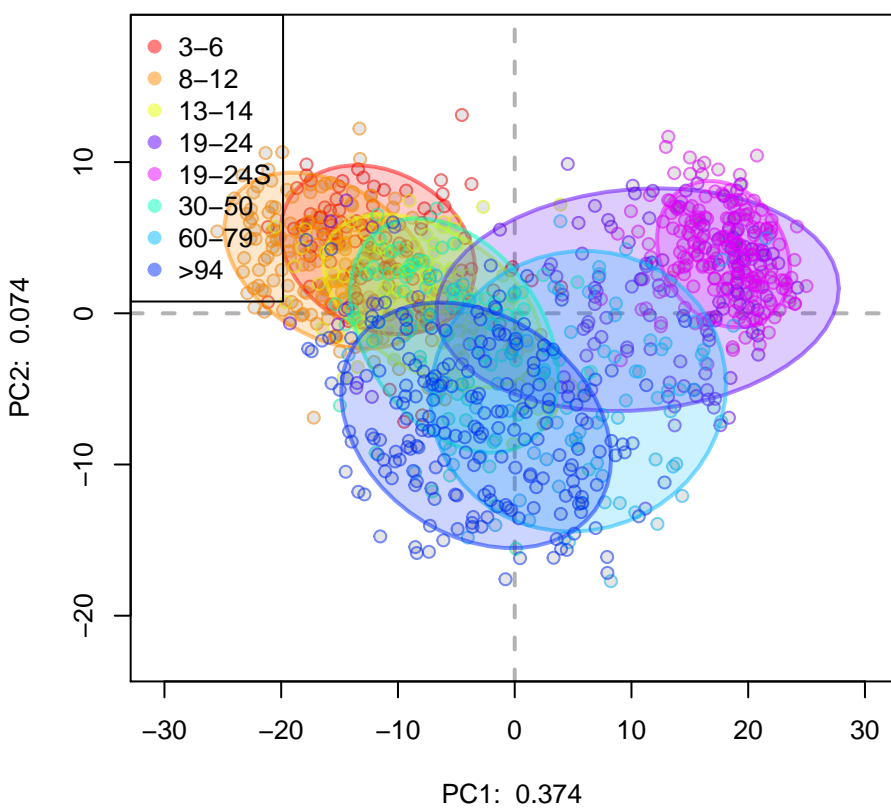

**D: OTU loadings**

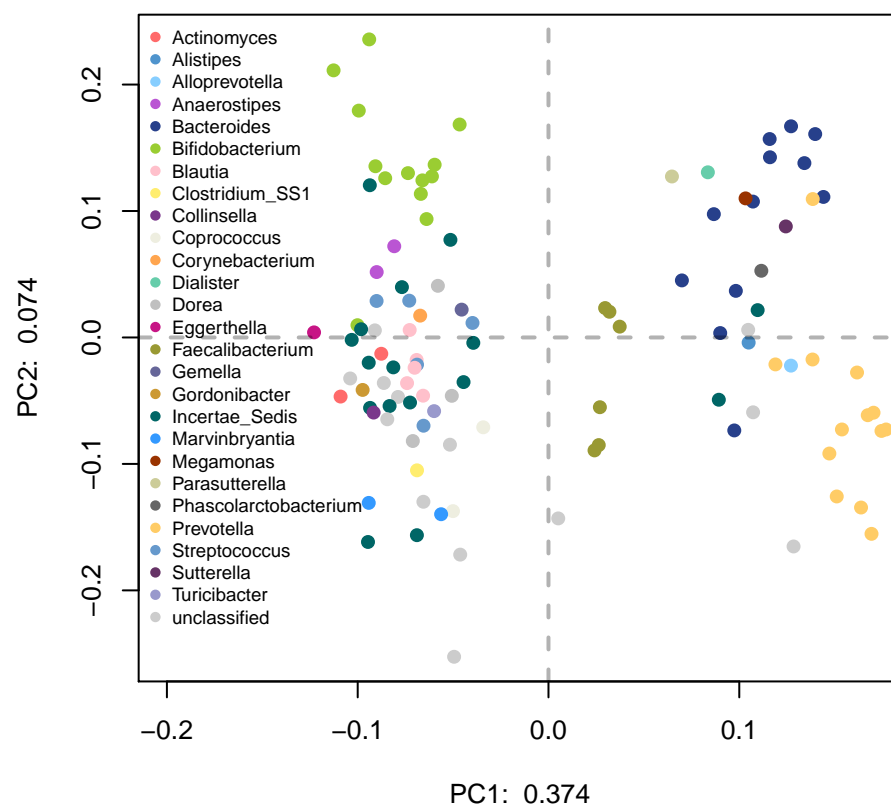

Supplement: FIG S2 [file sph005172374sf2.pdf]

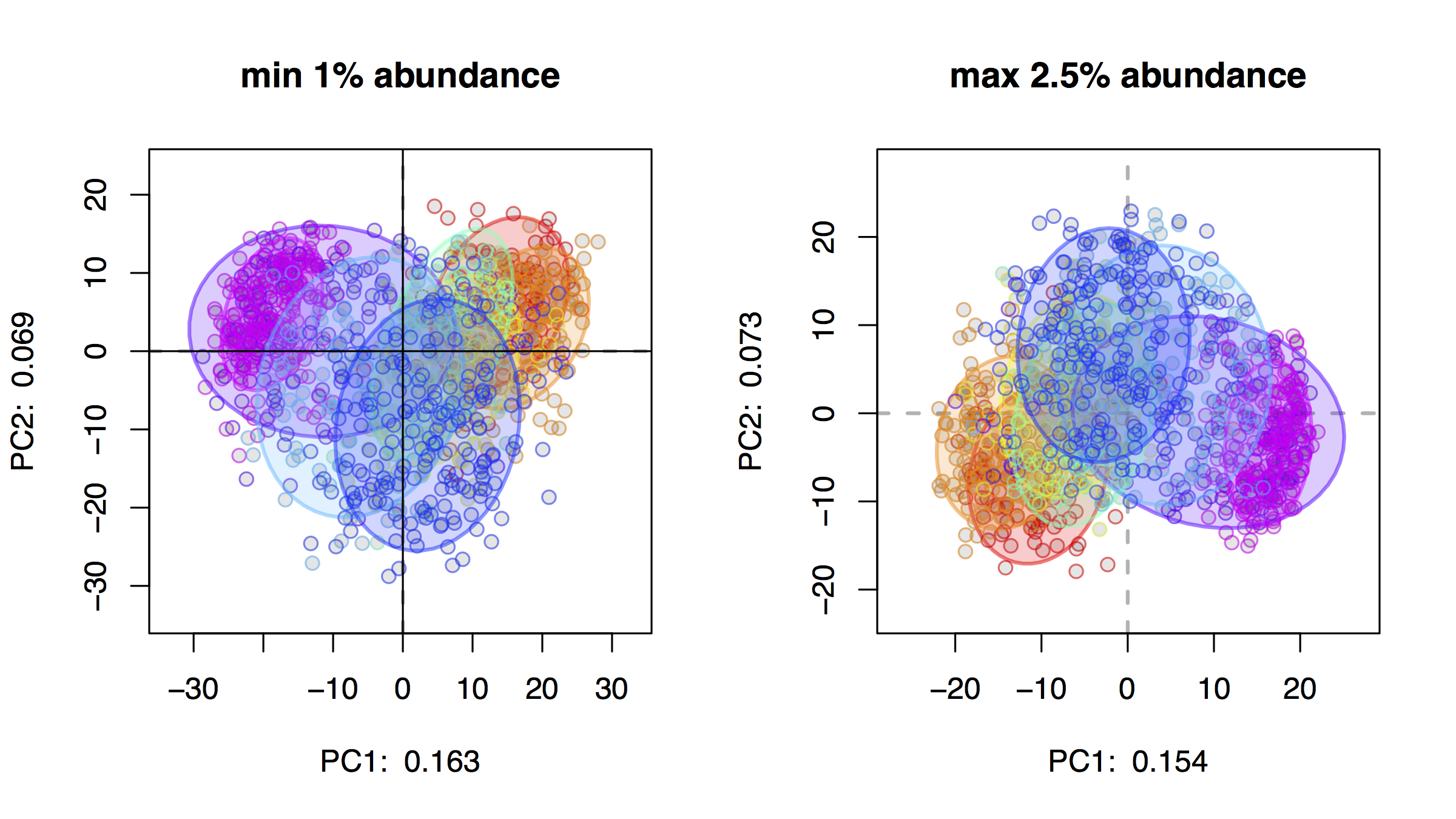

Supplement: FIG S3 [file sph005172374sf3.tif]

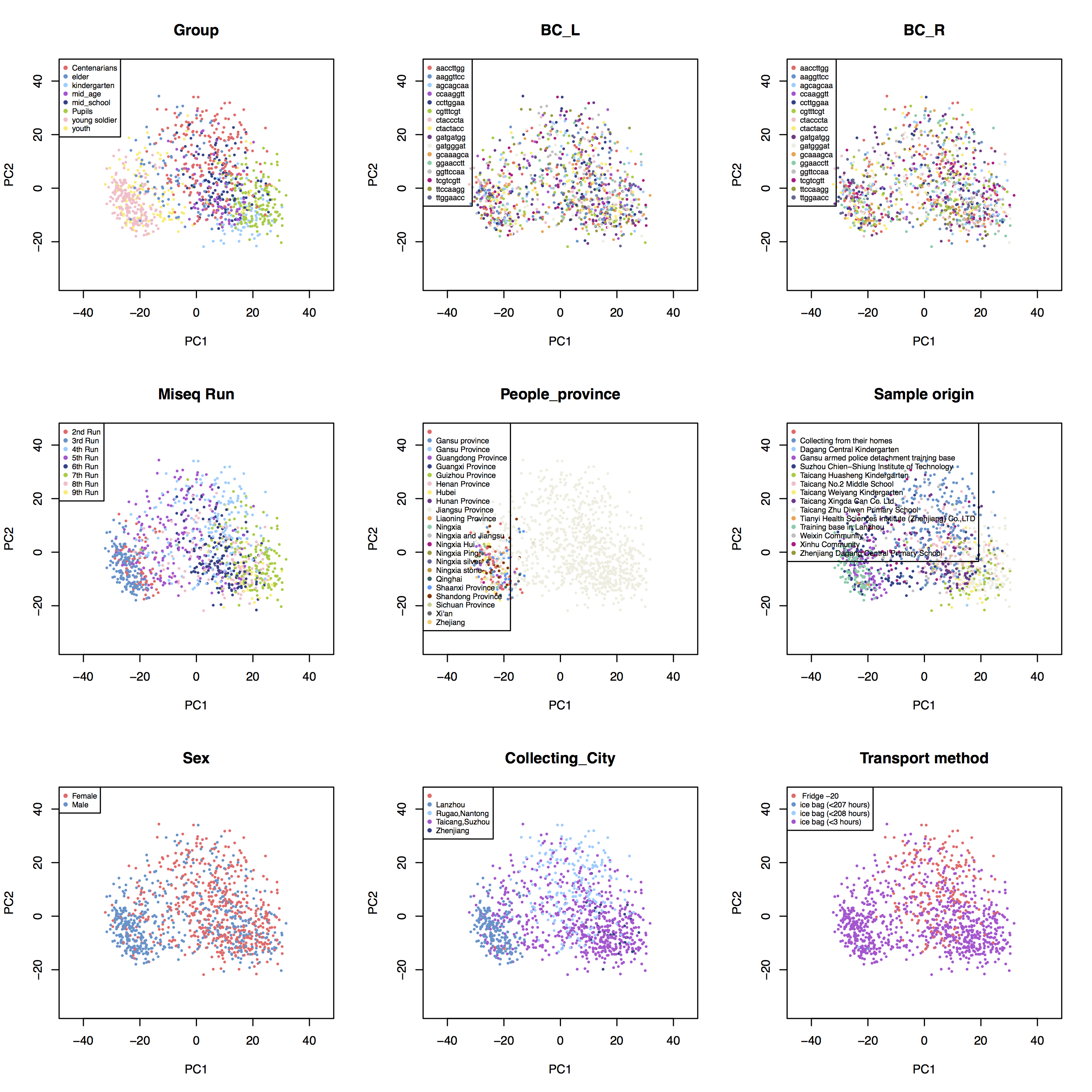

Supplement: FIG S4 [file sph005172374sf4.tif]

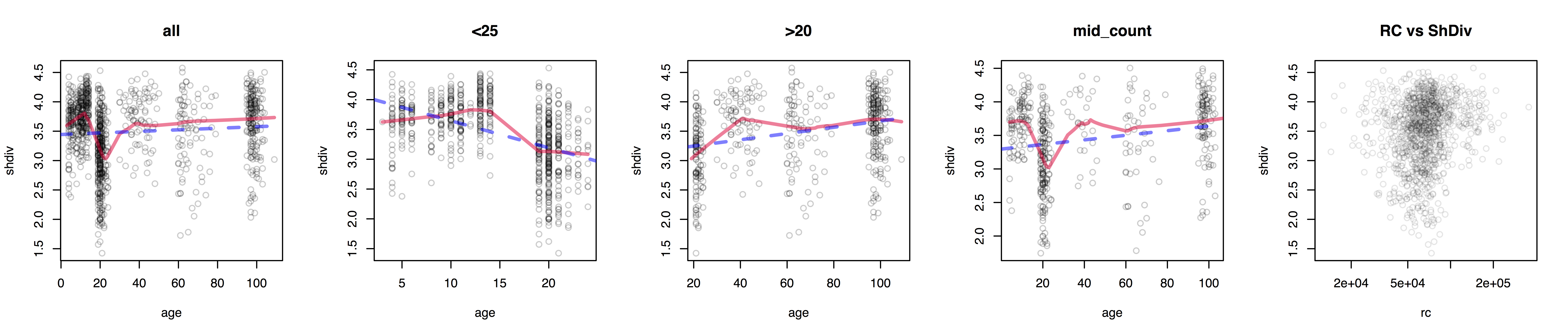

Supplement: FIG S5 [file sph005172374sf5.tif]

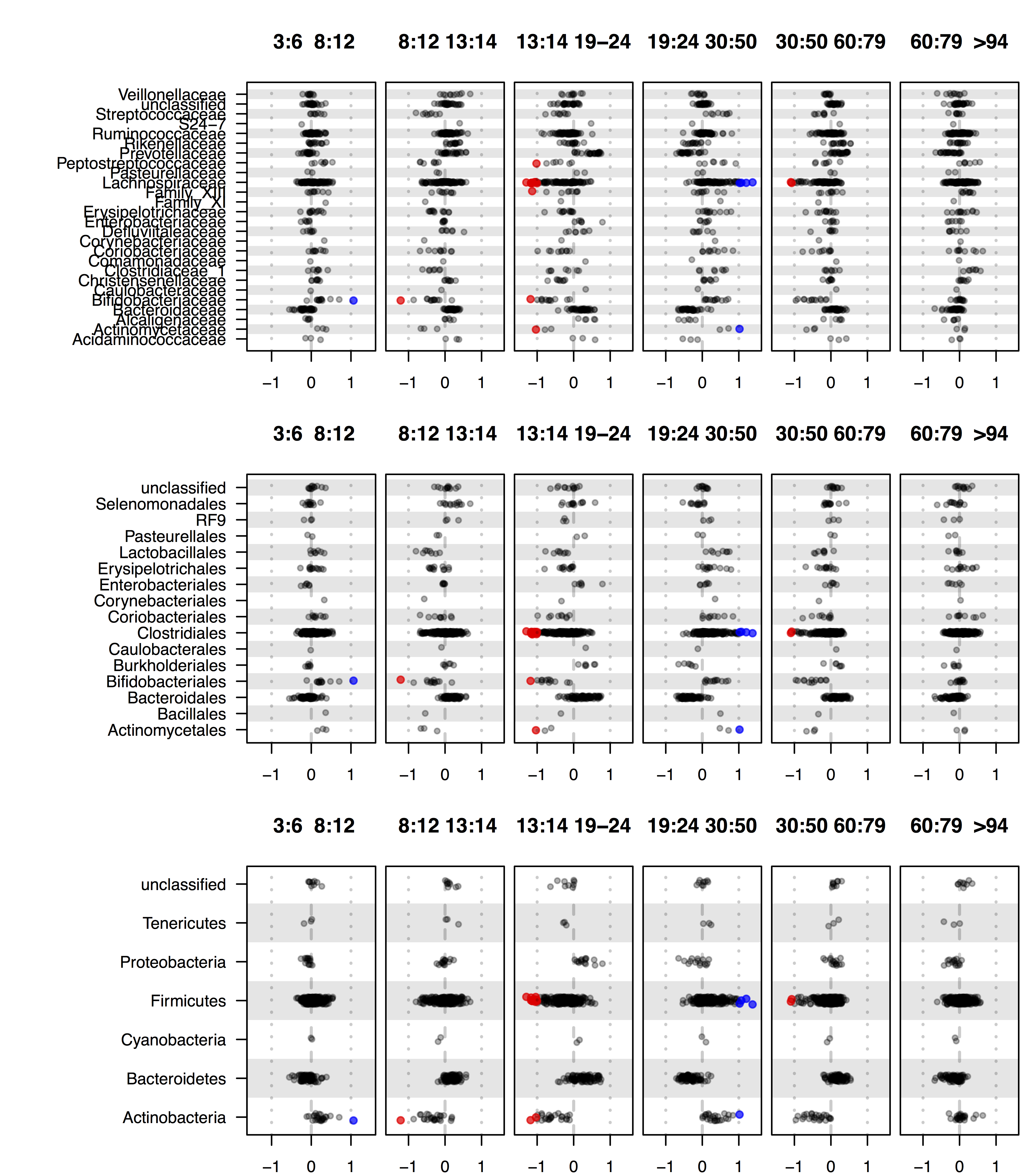

Supplement: FIG S6 [file sph005172374sf6.tif]

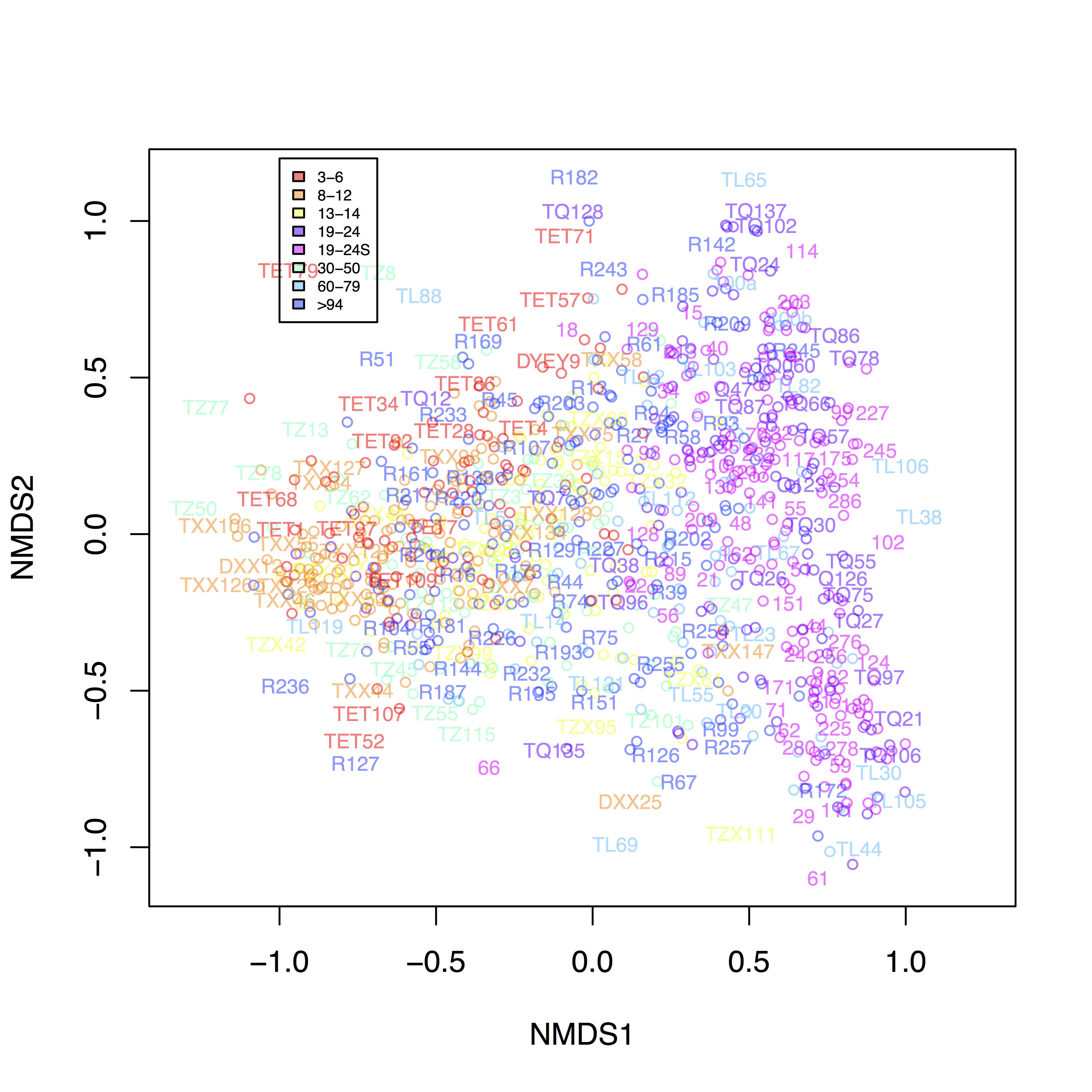

Supplement: FIG S7 [file sph005172374sf7.tif]
