# Supplementary material for: The Gut Microbiota of Healthy Aged Chinese Is Similar to That of the Healthy Young
Source: mSphere. 2017 Sep 27;2(5):e00327-17. doi: 10.1128/mSphere.00327-17 (PMC5615133; doi:10.1128/mSphere.00327-17)
Supplement: TABLE S1 [file sph005172374st8.docx]

Supplemental Table S1

| grpA | grpB | R2 | F.Model | P | MCD | IQR |
| --- | --- | --- | --- | --- | --- | --- |
| kin | pup | 0.025 | 6.6 | 0.001 | 36.8 | 34.4-39.2 |
| pup | mid | 0.053 | 15.159 | 0.001 | 38.3 | 34.5-40.8 |
| mid | you | 0.1 | 27.442 | 0.001 | 36.7 | 33.9-39.6 |
| mid | ys | 0.184 | 73.208 | 0.001 | - | - |
| you | ys | 0.036 | 12.843 | 0.001 | 36.3 | 32.9-40.7 |
| you | mage | 0.08 | 19.072 | 0.001 | - | - |
| ys | mage | 0.154 | 53.797 | 0.001 | 31.8 | 28.9-34.4 |
| mage | eld | 0.048 | 8.665 | 0.001 | 37.6 | 35.7-40.5 |
| eld | cent | 0.035 | 10.283 | 0.001 | 39.7 | 37.5-42.6 |
| cent | - | - | - | - | 43.4 | 41.5-45.2 |
