# Supplementary material for: The Gut Microbiota of Healthy Aged Chinese Is Similar to That of the Healthy Young
Source: mSphere. 2017 Sep 27;2(5):e00327-17. doi: 10.1128/mSphere.00327-17 (PMC5615133; doi:10.1128/mSphere.00327-17)
Supplement: TABLE S2 [file sph005172374st9.docx]

Supplemental Table S2

| OTU1 | genus1 | OTU2 | genus2 | E(rho) | intercept | slope | corr |
| --- | --- | --- | --- | --- | --- | --- | --- |
| 1452 | Bacteroides | 1 | Bacteroides | 0.7 | -8.33 | 0.98 | 0.82 |
| 891 | Prevotella | 2 | Prevotella | 0.82 | -3.47 | 0.78 | 0.87 |
| 1098 | Prevotella | 2 | Prevotella | 0.75 | -4.43 | 0.84 | 0.8 |
| 1219 | Prevotella | 2 | Prevotella | 0.76 | -4.95 | 0.79 | 0.83 |
| 1865 | Prevotella | 2 | Prevotella | 0.75 | -4.23 | 0.73 | 0.82 |
| 2859 | Prevotella | 2 | Prevotella | 0.81 | -5.94 | 0.85 | 0.91 |
| 2923 | Prevotella | 2 | Prevotella | 0.71 | -4.52 | 0.85 | 0.77 |
| 3464 | Prevotella | 2 | Prevotella | 0.81 | -5.86 | 0.9 | 0.89 |
| 3578 | Prevotella | 2 | Prevotella | 0.74 | -6.03 | 0.8 | 0.84 |
| 13 | Incertae_Sedis | 3 | Blautia | 0.74 | -0.74 | 0.89 | 0.75 |
| 15 | Incertae_Sedis | 3 | Blautia | 0.76 | -0.76 | 0.91 | 0.77 |
| 20 | Anaerostipes | 3 | Blautia | 0.73 | -1.25 | 0.94 | 0.75 |
| 36 | unclassified | 3 | Blautia | 0.72 | -0.39 | 0.78 | 0.73 |
| 3355 | unclassified | 3 | Blautia | 0.71 | -2.28 | 0.98 | 0.74 |
| 2611 | Bifidobacterium | 6 | Bifidobacterium | 0.69 | -3.61 | 0.71 | 0.79 |
| 3245 | Bifidobacterium | 6 | Bifidobacterium | 0.67 | -4.15 | 0.62 | 0.82 |
| 3430 | Bifidobacterium | 6 | Bifidobacterium | 0.8 | 0.42 | 0.82 | 0.83 |
| 2812 | Bacteroides | 9 | Bacteroides | 0.79 | -2.54 | 0.87 | 0.84 |
| 2340 | Streptococcus | 10 | Streptococcus | 0.75 | -0.96 | 0.7 | 0.8 |
| 15 | Incertae_Sedis | 13 | Incertae_Sedis | 0.7 | 2.21 | 0.7 | 0.7 |
| 20 | Anaerostipes | 13 | Incertae_Sedis | 0.7 | 1.6 | 0.75 | 0.71 |
| 3355 | unclassified | 13 | Incertae_Sedis | 0.66 | 0.94 | 0.75 | 0.67 |
| 20 | Anaerostipes | 15 | Incertae_Sedis | 0.7 | 1.5 | 0.75 | 0.7 |
| 22 | Blautia | 15 | Incertae_Sedis | 0.67 | 1.81 | 0.65 | 0.68 |
| 32 | Blautia | 15 | Incertae_Sedis | 0.7 | 1.46 | 0.65 | 0.71 |
| 36 | unclassified | 15 | Incertae_Sedis | 0.73 | 1.54 | 0.67 | 0.74 |
| 50 | Dorea | 15 | Incertae_Sedis | 0.66 | 0.4 | 0.65 | 0.68 |
| 3355 | unclassified | 15 | Incertae_Sedis | 0.8 | -0.35 | 0.91 | 0.82 |
| 1045 | Anaerostipes | 20 | Anaerostipes | 0.81 | -2.8 | 0.98 | 0.85 |
| 29 | Incertae_Sedis | 21 | Clostridium_SS1 | 0.66 | 2.31 | 0.63 | 0.67 |
| 32 | Blautia | 22 | Blautia | 0.65 | 1.96 | 0.64 | 0.66 |
| 49 | Incertae_Sedis | 29 | Incertae_Sedis | 0.75 | -0.51 | 0.81 | 0.77 |
| 36 | unclassified | 32 | Blautia | 0.71 | 1.96 | 0.7 | 0.72 |
| 3355 | unclassified | 36 | unclassified | 0.68 | 0.57 | 0.88 | 0.71 |
| 1466 | Veillonella | 69 | Veillonella | 0.72 | 0.51 | 0.75 | 0.78 |
| 1782 | Faecalibacterium | 105 | Faecalibacterium | 0.72 | -2.52 | 0.72 | 0.78 |
| 2656 | Faecalibacterium | 105 | Faecalibacterium | 0.75 | -5.64 | 1.02 | 0.87 |
| 1881 | Clostridium_SS1 | 292 | Clostridium_SS1 | 0.8 | 0.86 | 0.9 | 0.89 |
| 1098 | Prevotella | 891 | Prevotella | 0.67 | -0.2 | 0.9 | 0.76 |
| 1219 | Prevotella | 891 | Prevotella | 0.69 | -1.01 | 0.86 | 0.8 |
| 1865 | Prevotella | 891 | Prevotella | 0.7 | -0.67 | 0.81 | 0.82 |
| 2859 | Prevotella | 891 | Prevotella | 0.68 | -1.55 | 0.85 | 0.81 |
| 3464 | Prevotella | 891 | Prevotella | 0.72 | -1.32 | 0.96 | 0.85 |
| 1865 | Prevotella | 1098 | Prevotella | 0.65 | 0.04 | 0.64 | 0.76 |
| 2923 | Prevotella | 1098 | Prevotella | 0.8 | 0.06 | 0.95 | 0.9 |
| 3464 | Prevotella | 1098 | Prevotella | 0.69 | -0.5 | 0.77 | 0.8 |
| 3578 | Prevotella | 1098 | Prevotella | 0.73 | -1.51 | 0.79 | 0.87 |
| 3262 | Alistipes | 1167 | Alistipes | 0.79 | 0.22 | 0.94 | 0.87 |
| 1865 | Prevotella | 1219 | Prevotella | 0.69 | 0.49 | 0.76 | 0.82 |
| 3464 | Prevotella | 1219 | Prevotella | 0.7 | 0.07 | 0.89 | 0.84 |
| 3464 | Prevotella | 1865 | Prevotella | 0.69 | -0.21 | 0.95 | 0.83 |
| 2812 | Bacteroides | 2022 | Bacteroides | 0.65 | 4.03 | 1.09 | 0.85 |
| 2812 | Bacteroides | 2420 | Bacteroides | 0.67 | 2.43 | 0.82 | 0.78 |
| 3430 | Bifidobacterium | 2611 | Bifidobacterium | 0.78 | 4.73 | 0.97 | 0.89 |
| 3430 | Bifidobacterium | 2664 | Bifidobacterium | 0.66 | 6.33 | 1.07 | 0.83 |
| 3464 | Prevotella | 2859 | Prevotella | 0.7 | 0.56 | 0.93 | 0.86 |
| 3464 | Prevotella | 2923 | Prevotella | 0.65 | -0.3 | 0.69 | 0.76 |
| 3578 | Prevotella | 2923 | Prevotella | 0.72 | -1.4 | 0.75 | 0.87 |
| 3362 | Faecalibacterium | 3054 | Faecalibacterium | 0.67 | -5.09 | 0.88 | 0.8 |
| 3401 | Faecalibacterium | 3054 | Faecalibacterium | 0.7 | -6.73 | 1.06 | 0.88 |
| 3183 | Bacteroides | 3068 | Bacteroides | 0.69 | -6.23 | 1.03 | 0.84 |
| 3430 | Bifidobacterium | 3245 | Bifidobacterium | 0.66 | 5.82 | 1.06 | 0.82 |
| 3578 | Prevotella | 3464 | Prevotella | 0.68 | -0.76 | 0.8 | 0.84 |
