# Supplementary material for: The Gut Microbiota of Healthy Aged Chinese Is Similar to That of the Healthy Young
Source: mSphere. 2017 Sep 27;2(5):e00327-17. doi: 10.1128/mSphere.00327-17 (PMC5615133; doi:10.1128/mSphere.00327-17)
Supplement: TEXT S1 [file sph005172374s10.docx]

**SUPPLEMENTARY METHOD**

**(The exact workflow for 16S sequencing)**

Step 1: Download and de-compress the MiSeq reads. This is best done from the Il­lumina Basespace site, ask for access when you do your run. Place the reads into the reads/directory. Reads are compressed with 7Zip: from the command line:

7z e filename

gunzip XthRun_S1_L001_R1_00.fastq.gz

Step 2: Overlap the reads with pandaseq v2.5. An example command for this with a minimum overlap of 30 nucleotides is below. This command is appropriate for the V4 amplimers:

pandaseq -f XthRun_S1_L001_R1_001.fastq -r XthRun_S1_L001_R2_001.fastq -g ps_log.junk.txt -F -N -w reads/overlapped.fastq -T 2

Step 3: Run the workﬂow pipeline:

./workflow.sh name 0.97 V4EMB

3.1 extracting out the barcodes and primers associated with a particular samples.txt ﬁle. Output is a tabbed format ﬁle with the ﬁelds: read ID, sample ID primer sequence primer barcode q-score

$BIN/process_miseq_reads.pl $BIN samples.txt reads/overlapped.fastq $primer 8 0 $name T > $rekeyedtabbedfile

SPECIAL NOTE

COMBINE ALL SETS OF READS DESIRED INTO ONE FILE BEFORE PRODCEEDING WITH THE REMAINDER OF THE WORKFLOW. THIS WILL ENSURE THAT THE OTU PICKING, TAXONOMIC ASSIGNMENT, ETC WILL BE DONE ON AN HOMOGENEOUS DATASET.

3.2 Making a fasta ﬁle of all identical sequences (ISU), and an index of those sequences

$BIN/group_gt1.pl $rekeyedtabbedfile $name

3.3 Clustering at 97% identity using usearch (i.e., make OTU), also performs chimera ﬁlter singleton reads are excluded

$BIN/usearch -cluster_otus data_$name/groups_uclust.fa -otu_radius_pct 3 -otus data_$name/clustered_otus_usearch.fa

$BIN/usearch -usearch_global $groups_fa_file -db data_$name/clustered_otus_usearch.fa -strand plus -id 0.97 -uc $c95file

3.4 Regenerating the tabbed reads ﬁle with each read tagged as to its OTU and ISU group membership

$BIN/map_otu_isu_read_us7.pl $c95file $reads_in_groups_file $rekeyedtabbedfile > $mappedfile

3.5 Making two tables of counts in the analysis directory for OSU and ISU sequences gather the seed sequences for each OTU. Transpose the dataset for ease of import into QIIME

$BIN/get_tag_pair_counts_ps.pl $mappedfile $CUTOFF $name

$BIN/get_seed_otus_uc7.pl $c95file $groups_fa_file analysis_$name/OTU_tag_mapped.txt > analysis_$name/OTU_seed_seqs.fa

Rscript $BIN/OTU_to_QIIME.R analysis_$name

3.6 Using mothur to annotate the OTU sequences against the Silva database (v119)

mothur "#classify.seqs(fasta=analysis_$name/OTU_seed_seqs.fa, template=$TEMPLATE, taxonomy=$TAXONOMY, cutoff=70, probs=T, processors=4)"

$BIN/add_taxonomy_mothur.pl $TAX_FILE analysis_$name/td_OTU_tag_mapped.txt > analysis_$name/td_OTU_tag_mapped_lineage.txt
